# Research progress on the psychological burden and intervention measures in cancer patients

**DOI:** 10.3389/fpsyt.2024.1408762

**Published:** 2024-06-13

**Authors:** Han-Qi Wang, Hao Lin, Bing Liu

**Affiliations:** ^1^ State Key Laboratory of Oral & Maxillofacial Reconstruction and Regeneration, Key Laboratory of Oral Biomedicine Ministry of Education, Hubei Key Laboratory of Stomatology, School & Hospital of Stomatology, Wuhan University, Wuhan, China; ^2^ Department of Oral & Maxillofacial Head Neck Oncology, School & Hospital of Stomatology, Wuhan University, Wuhan, China

**Keywords:** cancer, psychological burden, depression, demoralization, predictive model

## Abstract

In the past 40 years, the gradually increasing incidence and mortality rates of malignant tumors have severely impacted the quality of life of patients, bringing significant physical and psychological burdens and becoming an increasingly serious social issue. With the development of medical standards, new methods for cancer detection and treatment have been continuously proposed. Although it has been proven that cancer is related to increased psychological burden and suicidal behaviors in patients, current research on the psychological burden caused by cancer is insufficient. Clinicians often overlook the psychological health issues of patients while treating their physical diseases. Considering the high incidence of cancer, this review will outline the psychological burdens of cancer patients worldwide in recent years and its high-risk factors. Moreover, this review will summarize the common methods for evaluating psychological burdens, present current predictive models and treatment methods for the psychological burden of cancer patients, aiming to provide a research basis and future direction for the timely and accurate assessment of the psychological burden in cancer patients.

## 1 Introduction

Nowadays, the incidence and mortality rates of cancer worldwide are showing a continuous upward trend, becoming a severe social issue in the context of increasing average lifespans. There are approximately 19 million to 20 million new cancer cases annually worldwide, with deaths reaching around 10 million. In China, the annual number of new cancer cases among people over 60 years old is about 2,790,000 (1, 2). Additionally, the World Health Organization (WHO) predicts that by 2045, the global number of new cancer cases will reach 32.6 million, with an estimated 16.9 million deaths. Due to the rising incidence and mortality rates of cancer, researchers have invested considerable resources in related fields such as cancer diagnosis, treatment, prevention, and prognosis. Personalized treatment plans have been proposed based on different cancer origins, disease stages, and genetic phenotypes, significantly improving the 5-year survival rate of cancer patients (3). However, compared to cancer diagnosis and treatment, the important health issue of cancer patients’ psychological burden has not been adequately studied. Particularly in developing countries, clinicians often neglect patients’ mental health, leading to a situation of “treating the body but not the mind” (4).

Since the 1970s, psycho-oncology, as a new interdisciplinary field, has become part of cancer treatment. Being diagnosed with cancer often causes patients to lose their life goals and changes their self- and social perceptions, leading to mental distresses such as fear, anxiety, depression, and demoralization, especially in patients with underlying mental illnesses (5). Studies indicate that 35%-52% of cancer patients experience a high psychological burden (6). Excessive psychological burden can induce a series of symptoms and signs, including insufficient rest, dry mouth, and difficulty breathing, which not only affect the prognosis of cancer but may even lead to self-injurious and suicidal behaviors in patients (7). Therefore, screening for the psychological burden of cancer patients as part of routine cancer diagnosis and treatment to implement timely and effective psychological interventions can help improve the prognosis of cancer patients and prevent extreme behaviors.

**Figure 1:**
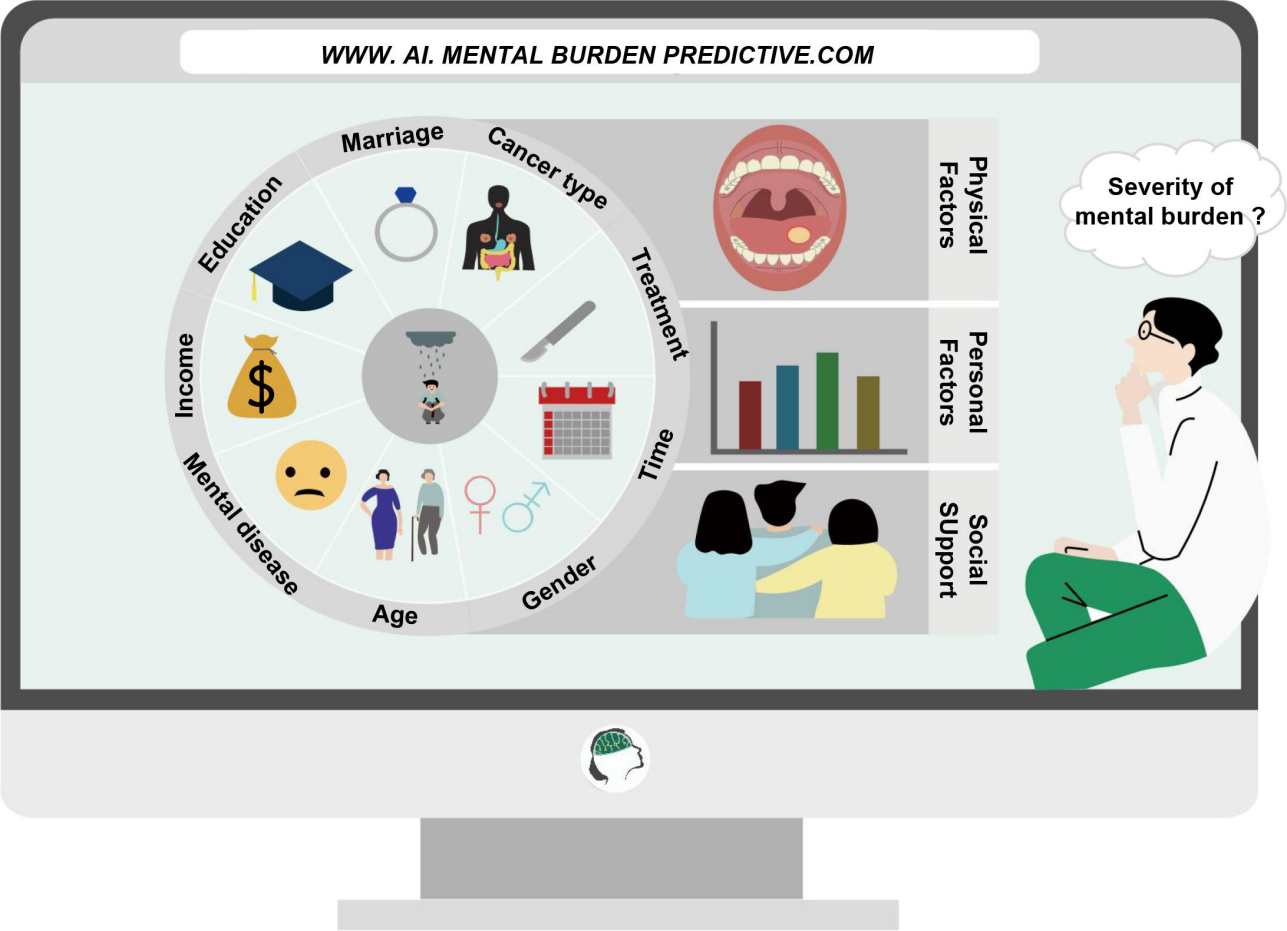
Monitoring and intervention methods for the psychological burden of cancer patients in the new era. Using an artificial intelligence platform, a predictive model of psychological burden is constructed based on the risk factors identified in cancer patients. Clinical practitioners can achieve rapid assessment and timely intervention for the psychological burden of cancer patients based on the actual conditions of patients and with the aid of artificial intelligence.

This review outlines the common psychological burdens and corresponding symptoms in patients diagnosed with cancer, introduces common high-risk factors affecting the mental health of cancer patients; at the same time, we summarize the current common assessment methods and predictive measures, treatment methods for the psychological burden in cancer patients, and discuss the future direction of psycho-oncology and the obstacles to be overcome (**Figure 1**).

## 2 Overview of psychological burden in cancer patients

Cancer patients often present psychological disorders after diagnosis, during and after the treatment such as adjustment disorders, anxiety, affective disorders, demoralization, and post-traumatic stress disorder. In this section, we will summarize and discuss four common psychological burdens experienced by cancer patients: adjustment disorders, anxiety, depression, and demoralization. Additionally, we will introduce changes in the psychological states of patients during the unique period of the COVID-19 pandemic.

### 2.1 Adjustment disorders

In 2018, WHO updated the definition of adjustment disorders as adverse reactions to stressors (such as a cancer diagnosis, setbacks in family and career, etc.) within one month of encountering the stress (8). Adjustment disorders are usually temporary, and people gradually return to a healthy state over time. However, if this state persists, it can induce more severe psychological issues in the patient, such as depression and anxiety, and even lead to suicide (9). Studies show that health-related stressors are significant risk factors for inducing adjustment disorders, which are common among cancer patients, with 13%-30% possibly experiencing them (10–12). Evaldas et al. conducted a 12-month follow-up on prostate cancer patients, finding that the incidence of adjustment disorders was 15% at the time of cancer diagnosis, 13% three months after diagnosis, and 3% twelve months after diagnosis, showing a declining trend over time (13). It is noteworthy that the incidence of adjustment disorders appears to vary among different types of cancer. According to a study by Dai et al., only 6% of 6,392 newly diagnosed breast cancer patients exhibited adjustment disorders (14). In contrast, 14% of adolescent cancer patients (15) and 16.5% of head and neck cancer patients (16) were found to have adjustment disorders, which is significantly higher than in breast cancer patients. The tumor-specific nature of adjustment disorders may be related to factors such as the treatment modalities, age at diagnosis, and prognosis of the tumor (13–17). Additionally, the variety of methods used to assess and diagnose adjustment disorders can lead to significant differences in the reported incidence rates among different studies (18).

### 2.2 Anxiety

Anxiety is defined as worry about future misfortune and danger, accompanied by restlessness and tense somatic symptoms. Anxiety can cause symptoms including palpitations, sweating, sleep disorders, and seeking comfort. Although anxiety is common in cancer patients, some exhibit overwhelming symptoms, potentially leading to an overestimation of negative cancer prognoses and severely affecting daily life (7). Naser et al. conducted psychological assessments on 1,011 cancer patients (including 612 outpatients and 399 inpatients), finding an anxiety symptom incidence of approximately 19.1%-19.9%, occurring more frequently among inpatients (16). The systematic review of Mohammad et al. showed that cancer patients’ average death anxiety score was 6.84, with Asians and young women with breast cancer showing more pronounced death anxiety (19). Sleep disorders, a common symptom of anxiety, affect 33%-50% of cancer patients, and many cancer survivors report it as one of their most common current problems (20). A follow-up of 2,611,907 cancer patients indicated that their anxiety symptoms were positively correlated with specific mortality rates and negatively correlated with cancer survival rates. Additionally, anxiety disorders are positively correlated with cancer incidence (21).

### 2.3 Depression

Previous research indicates that the prevalence of depression in cancer patients is 2–3 times higher than in the general population, and patients’ depression symptoms often coexist with anxiety and adjustment disorders (16). In a cross-sectional study, 21.5% of cancer patients undergoing radiotherapy or chemotherapy exhibited depressive symptoms (22). Nineteen percent of long-term cancer survivors have moderate to severe depression symptoms, with higher levels observed in women and younger patients in their working years. There is no significant difference in depression rates between 5-year and 10-year survivors (23). Breast cancer patients, in particular, show a higher proportion of depression symptoms, up to 66%-68.6%, with long-term survivors having higher depression scores five years after diagnosis compared to 40 weeks (24–26). A cross-sectional study in China indicated that 65.21% of oral cancer patients also show depression symptoms (27). Huang et al.’s study on the psychological burden of non-small cell lung cancer patients in northern China showed that 38.3% of patients exhibit depression symptoms, compared to 10% in the general population (28). The systematic review of Oliver et al. on prostate cancer patients revealed that only 5.81% had depression, 17.07% exhibited depressive symptoms, and 9.85% had suicidal thoughts, with a suicide rate of 47.1 per 100,000 person-years (29).

### 2.4 Demoralization

Demoralization is a manifestation of psychological stress, typically characterized by helplessness, hopelessness, and loss of meaning and goals in life, varying from depression to despair. It is commonly found in patients with progressive diseases and cancer (30). In mainland China, clinicians often conflate demoralization with depression, but unlike the lack of pleasure in depression, patients with demoralization syndrome can still experience happiness. Reports suggest that demoralization is more common than depression in cancer patients and more closely associated with suicidal intentions (31, 32). The systematic review of Lin et al. indicated that about 25.98% of cancer survivors experience demoralization, with breast cancer patients scoring higher than others (32). A cross-sectional study in China found that over half of the patients in Xiamen showed signs of demoralization which may be related to living standards, family income, etc. (33). Demoralization often occurs in advanced cancer patients. Garzón’s study indicated that 53.4% of advanced cancer patients exhibited demoralization, with 16% moderate and 37.4% severe (34). Notably, not only advanced cancer patients but also their family members show signs of demoralization. Reports suggest that the family members of terminal cancer patients have an average demoralization score of 29.04, with 39% of caregivers experiencing severe demoralization (35).

### 2.5 Psychological burden of cancer patients during the COVID-19 pandemic

Since late 2019, the COVID-19 pandemic has swept across the world, with most countries implementing lockdown measures during the outbreak. Changes in lifestyle not only affected physical health but also significantly increased psychological burden. Reports indicate that cancer patients are a high-risk group for COVID-19 infection, with depression, anxiety, insomnia, post-traumatic stress disorder being the main psychological issues during the pandemic (36). Wang et al. conducted a cluster sampling study to analyze the psychological burden of cancer patients during the pandemic, finding that among 6,213 patients, 23.4% exhibited depression, 17.7% anxiety, 9.3% post-traumatic stress disorder, and 13.5% hostility (37). Cancer-related treatments, such as chemotherapy, cause immunosuppression in patients, increasing the risk of COVID-19 infection. A study investigating the psychological state of breast cancer patients during and after chemotherapy showed that 44.4% of them exhibited significant levels of anxiety, 41.7% insomnia symptoms, and 52.8% fear of cancer recurrence, with most (63.9%) experiencing at least one COVID-19 related stressor (38). A prospective trial investigating the psychological burden of lymphoma patients found that 36% of the patients exhibited anxiety symptoms, 31% depression symptoms, 36% post-traumatic stress disorder, with the fear of hospital-acquired COVID-19 infection and treatment interruption being their main concerns (39).

## 3 Factors influencing the psychological burden of cancer patients

The psychological burden of cancer patients is influenced by multiple factors. This section summarizes the high-risk factors for psychological issues in cancer patients from three aspects: physical factors of the patient, personal factors, and social factors.

### 3.1 Physical factors

Factors such as the primary site of the tumor, treatment, clinical staging, changes in appearance, and functional impairment in cancer patients have all been proven to correlate with the severity of their psychological burden. The systematic review by Riedl et al. pointed out that physical factors like the stage of the disease, symptoms caused by cancer treatment (such as fatigue, postoperative adverse reactions, uroclepsia), patient’s comorbidities, severity of pain, and adjuvant therapy, can increase the risk of depression in patients (40). A cross-sectional study compared the self-assessed physical and psychological health status of ovarian cancer patients with recurrence to those without recurrence. The results showed that 59.5% of recurrent patients reported that psychological factors limited their activities, which was significantly higher than the 15.8% of patients without recurrence (41). Studies have indicated that cancer recurrence is a risk factor for emotional disorders (OR=40), adjustment disorders (OR=3.51), and anxiety (OR=6.58) in cancer patients (42). Additionally, based on the SEER database, Rieke et al. noted that the staging of the disease in head and neck cancer patients also impacts their psychological burden. Among those with depression, 19.2% were in the advanced stages of the disease, compared to only 13.3% of non-depressed cancer patients in the advanced stages of head and neck cancer (43). The impact of psychological burden in cancer patients exhibits a clear cancer-type specificity. Statistics show that inpatients are more likely to experience psychological issues compared to outpatients. Among inpatients, depression symptoms are more common in bladder cancer patients, while lung cancer patients exhibit more common anxiety symptoms; among outpatients, breast and prostate cancer patients are more likely to experience depression and anxiety symptoms, respectively (16). The extent of psychological burden in different types of cancer patients also changes with the duration since diagnosis. Manne et al. pointed out that the psychological resilience of patients with gynecological cancers decreases over time from diagnosis, with an increasing psychological burden (44); whereas in liver cancer patients, the psychological burden decreases over time from diagnosis (45). This phenomenon is also related to the different physical burdens of various types of cancer at different times post-diagnosis. In lung and colorectal cancer, significant complications such as sleep disturbances and pain mainly occur within the first 16 months after diagnosis; in contrast, in breast cancer, over half (61%) of long-term survivors diagnosed for more than five years’ experience severe pain, fatigue, and sleep disturbances, exacerbating their psychological burden (46).

Malignancies such as head and neck cancer, due to their unique tumor site and surgical treatment modality, have unique risk factors on the psychological burden of patients. For example, concerning changes in appearance, 13%-20% of head and neck cancer patients experience distress related to their appearance, which is positively correlated with the occurrence of depression symptoms (47). Additionally, Lee et al. conducted a cross-sectional study on 52,164 head and neck cancer patients, finding that locations such as the nasopharynx and hypopharynx, due to their severe impact on patients’ swallowing and respiratory functions, lead to more frequent psychological issues, with 29.9% of patients experiencing psychological problems, an increase of about 9% compared to before their diagnosis (48). Similar to patients with head and neck cancer, elderly prostate cancer patients who underwent prostatectomy show a significant positive correlation between postoperative psychological burden and uroclepsia (49). The use of androgen deprivation therapy in the treatment of prostate cancer also increases the risk of depression (50).

### 3.2 Personal factors

Demographic factors such as gender, age, and religious beliefs are common predictors in research on the psychological burden of cancer patients. Reports indicate that being female is a risk factor for psychological issues in cancer patients, with male patients having a 71% and 59% lower risk of developing depression and anxiety symptoms, respectively, compared to females (45, 51). However, some studies suggest that compared to the same age group in the general population, male cancer patients exhibit a significantly higher proportion of symptoms like depression, anxiety, and difficulty breathing than female patients (52). Regarding the relationship between age and psychological burden in cancer patients, the correlation exists, but the specific outcomes are not definite: many studies show that younger patients are more likely to experience psychological issues (53–55). However, other research indicates that cancer patients over 85 years old are more likely to suffer from depression than younger patients, possibly due to more severe complications in older adults; whereas young cancer patient groups exhibit a higher frequency of anxiety symptoms compared to the elderly, possibly related to better emotional regulation in older adults (56). Research has shown that religious beliefs can promote the mental health of cancer patients and reduce their pain (57). Tsaras et al.’s cross-sectional study pointed out that non-Orthodox believers among breast cancer patients are a predictor of psychological issues (58). Guan et al. found that in Malaysia, the depression and anxiety of cancer patients are negatively correlated with private participation in religious activities. Patients can use their faith to combat the shock of a cancer diagnosis, reducing thoughts of pain and extreme behavior (59). Evangelos et al. noted that religious beliefs are closely linked to the psychological resilience of cancer patients and are an important predictor of resilience. Many late-stage breast cancer patients seek psychological comfort at the end of their lives by believing in God’s healing powers. Religious faith can provide social support for cancer patients, helping them adjust their mindset promptly and face the disease positively (60).

The psychological burden of cancer patients, apart from being influenced by demographic factors, is also related to pre-diagnosis mental health issues. Bouras et al.’s study using logistic regression analysis showed that patients undergoing esophageal and gastric cancer surgery with preoperative mental illness have a significantly increased risk of postoperative depression or anxiety, with an odds ratio (OR) of 6.73 (61). Saboonchi et al. pointed out that pre-surgery life stress in breast cancer patients also increases the likelihood of postoperative anxiety and depression (with an OR of 3.53) (62). Conversely, patients with optimistic and easy-going traits have a higher quality of life after being diagnosed with cancer, thus reducing their psychological burden (63–65).

### 3.3 Social factors

Social support refers to the care and assistance perceived and felt by an individual, provided by their social network and close partners. It is generally divided into instrumental support (concrete assistance) and emotional support (psychosocial help). It has been reported that both effective information provision and emotional support can alleviate the psychological burden of cancer patients (66).

On one hand, in terms of effective information acquisition: a psychological study of newly diagnosed esophageal and gastric cancer patients showed that the time interval between cancer diagnosis and decision on treatment plan for patients without a history of mental disorders is related to the frequency of psychiatric hospital visits within a year after diagnosis. A longer wait for treatment (30–60 days) compared to timely determination of treatment plans (18–29 days) significantly increases the risk of psychological disorders (67). Most cancer patients begin seeking disease-related information such as cancer treatment strategies and common treatment adverse events after diagnosis, to achieve timely and accurate treatment of cancer. The acquisition of this effective information is usually related to the professionals and volunteers that cancer patients can access. Consequently, researchers have investigated the economic and social factors like place of residence, economic conditions, and education level of cancer patients in relation to their psychological burden. Reports indicate that being a rural resident, receiving less than 8 years of education, low social support, and low income are associated with OR of depression symptoms of 1.14, 1.97, 2.84, and 4.41, respectively. Additionally, high social support is closely related to a higher quality of life for patients (42, 49, 68, 69).

In addition, interpersonal relationships of cancer patients also impact their psychological burden. Reports suggest that the psychological burden of elderly cancer patients is significantly related to their marital status and cohabitants. Widowed patients bear a higher psychological burden, while those living with spouses and children have the lowest burden. Patients living only with their children show the highest psychological burden (70). In malignancies like glioma, colorectal, and breast cancer, factors such as being single, divorced, or widowed have been proven to be independent risk factors for depression (71–73). Notably, Shi et al. points out that in breast cancer patients, high-quality intimate relationships (dependence relationships formed between cancer patients and their spouses in coping with the disease) may be detrimental to post-traumatic growth (β= -0.309 in the structural equation model). This might be because spouses of patients with high-quality intimate relationships tend to hide or deny cancer-related worries, increasing mutual suspicion (74).

## 4 Modern assessment and intervention for psychological burdens in cancer patients

For cancer patients receiving treatment, the timely and accurate identification of their psychological problems by oncologists is a prerequisite for intervening in their psychological burden. Furthermore, with the continuous development of computer technology and artificial intelligence in medical assistance, monitoring of psychological issues in cancer patients has become more convenient and efficient. This section will focus on the current research progress in traditional assessment methods and monitoring tools for psychological burden in cancer patients and summarize common intervention measures from both personal and hospital perspectives.

### 4.1 Assessment methods for psychological burden in cancer patients

Unlike organic diseases, which can be diagnosed using imaging studies, physiological biochemical tests, and pathological examinations, the assessment of psychological burden in the cancer population is mainly based on scales (**Table 1**). The Center for Epidemiologic Studies Depression Scale (CES-D) is widely used to assess depression symptoms in cancer patients, covering 20 items across four dimensions: depressive affect, positive affect, somatic symptoms, and interpersonal relationships. Several studies have demonstrated good internal consistency and repeatability of the CES-D scale in assessing depression symptoms in various cancer patient populations (75, 76, 100). The Patient Health Questionnaire-9 (PHQ-9) includes 9 items and is more convenient and time-saving for cancer patients compared to longer scales. It has been reported that the optimal cut-off point for the PHQ-9 scale is ≥9, with a sensitivity of 88% and specificity of 80% (79). The State-Trait Anxiety Inventory (STAI) is a 40-item self-report scale used to assess and differentiate between state and trait anxiety; its revised version, STAI-Y, reduces overlap with depression. Studies have shown that the STAI-Y scale with a cut-off point >23 has a sensitivity of 94.3% and specificity of 93.6% (101). The Hospital Anxiety and Depression Scale (HADS) consists of 14 items, including two subscales for anxiety and depression (HADS-A, HADS-D), mainly used for screening anxiety and depression symptoms in hospital patients in non-psychiatric settings (102, 103). According to studies, the optimal cut-off point for HADS-A is >9, with a sensitivity of 83.2% and specificity of 80.5%; for the HADS-D scale, the optimal cut-off point is >7, with a sensitivity of 72.9% and specificity of 79% (103). However, Hartung et al. compared the HADS and PHQ-9 scales and found that their accuracy in diagnosing severe depression is comparable and lower than previously reported. These scales have limited screening capacity for depression compared to standardized diagnostic interviews, potentially leading to false-positive cases (104). Moreover, for the assessment of demoralization in cancer patients, the common scale used is the revised Demoralization Scale (DS-II). The DS-II scale has 16 items covering two subscales, including meaning and purpose, distress and coping ability. The Cronbach’s α coefficient of the DS-II scale is reported to be 0.93, indicating excellent internal consistency (92). It is important to note that detecting psychological problems in cancer patients today is quite challenging, as cancer symptoms often overlap with psychological issues causing fatigue, loss of appetite, sleep disturbances, etc. Additionally, due to shame in facing their psychological issues and an attitude of protecting privacy, patients may not truthfully fill out their psychological conditions in surveys (105, 106). Therefore, seeking new ways to screen patients’ psychological burdens is crucial to accurately and timely intervene and treat their psychological distress.

**Table 1:** Psychological Burden Assessment Scales for Cancer Patients.

Scale Name	No. of Items	Internal Consistency (α)	Symptom Severity	Common Cancer Types	References
Depression					
CES-D	20	>0.85	>16 indicates depression	1. Breast cancer; 2.Colorectal cancer; 3. Oral (Pharyngeal) Cancer; 4. Lung Cancer.	(75–78)
PHQ-9	9	0.78	5–9: Mild to Moderate Depression; >10: Moderate to Severe Depression.	1. Breast cancer; 2.Gastric cancer; 3. Skin cancer.	(79–81)
HADS-D	7	0.85	8–10: Suspected Depression; >10: Depression.	1. Breast cancer; 2. Lung cancer; 3. Gastric cancer; 4. Prostate cancer; 5. Hematologic malignancies.	(82–84)
BDI-II	21	0.91	14–19: Mild Depression; 20–28: Moderate Depression; 29–63: Severe Depression.	1. Breast cancer; 2. Lung cancer; 3. Prostate cancer; 4.Gastrointestinal cancer; 5. Gynecological cancer.	(85, 86)
DASS-21 Depression Subscale	7	0.91	10–13: Mild Depression; 14–20: Moderate Depression; 21–27: Severe Depression; >28: Extremely Severe Depression	1. Breast cancer, 2. Prostate cancer, 3. Colorectal cancer.	(87, 88)
Anxiety					
HADS-A	7	0.86	8–10: Suspected Anxiety; >10: Anxiety.	1. Breast cancer; 2. Lung cancer; 3. Gastric cancer; 4.Prostate cancer; 5.Hematologic malignancies.	(82–84)
GAD-7	7	0.83–0.92	5–9: Mild Anxiety; 10–14: Moderate Anxiety; >15: Severe Anxiety.	1. Hematologic malignancies; 2. Colorectal cancer; 3. Bladder cancer; 4. Gastric cancer.	(16, 89)
STAI-Y	40	0.88–0.9	>60: Increased Anxiety	1. Breast cancer; 2. Bladder cancer.	(90, 91)
DASS-21 Anxiety Subscale	7	0.7	8–9: Mild Anxiety; 10–14: Moderate Anxiety; 15–19: Severe Anxiety; >20 Extremely Severe Anxiety.	1. Breast cancer; 2. Prostate cancer; 3. Colorectal cancer.	(87, 88)
Demoralization					
DS-II	16	0.88–0.93	<7: Low Demoralization 8–17: Moderate Demoralization >18: High Demoralization	1. Advanced cancer; 2. Breast cancer; 3. Prostate cancer; 4. Head and Neck cancer	(92–94)
Bipolar Disorder					
MDQ	13	0.84	>7: Bipolar Disorder	Prostate cancer	(95)
Post-Traumatic Stress					
PCL-C	17	0.91	>17:PTSD	1. Breast cancer; 2. Head and Neck cancer; 3. Lymphoma	(96, 97)
IES-R	22	0.84–0.92	>33:PTSD	1. Breast cancer; 2. Lymphoma; 3. Central Nervous System malignancies	(98, 99)

### 4.2 Artificial intelligence predictive models for psychological burden in cancer patients

With the development of computer technology, AI is increasingly applied in the diagnosis and treatment of tumors. However, in the identification and prediction of the psychological burden of cancer patients, AI is still in its infancy, and the field holds significant research value. Susheela et al., based on the PHQ-9 depression scale and GAD-7 anxiety scale, assessed the risk factors of psychological burden in female ovarian cancer patients using common machine learning techniques such as random forests, linear support vector machines, and artificial neural networks, and constructed predictive models for depression and anxiety in ovarian cancer patients. The study showed that the accuracy rates of the linear support vector machine for depression and anxiety were 91.52% and 93.78%, respectively (107). In addition to using scales, AI can also assess patients’ psychological burdens through means such as facial expressions (e.g., reduced facial activity, avoiding eye contact) and voice data (e.g., slower speech, pauses in speaking, increased usage of first-person pronouns) (108). Chen et al. developed a method to identify the psychological burden of cancer patients using a machine learning model based on facial expression features, verifying that the model’s accuracy could reach 82%-93.3%, demonstrating the immense potential and significant role of patient facial expressions in predicting psychological burden (109). To reduce the tedium of patients using questionnaires to assess psychological burden during medical visits, Zhang et al. proposed a cohort study plan to recognize the psychological burden of adolescent and young cancer patients based on voice recognition. The project plans to use the Ellipsis Health voice tool (EH) for a 6-month mental stress monitoring of patients aged 15 to 26. Unfortunately, the results of this project have not yet been made public (110). Additionally, the structure and function of the brain are important biomarkers for identifying mental disorders, so AI processes and analyzes patients’ neuroimaging data (such as MRI, EEG) for predictive purposes in mental illness (111, 112). Research indicates that in resting-state functional MRI and diffusion tensor imaging results of rectal cancer patients, those with depressive tendencies show impairment in the functional and structural networks of the brain (113). More importantly, unlike patients’ reluctance to reveal their true condition when using traditional psychological assessment scales, according to Lucas et al.’s study, patients are more willing to self-disclose when interacting with computer-simulated humans, without worrying about the negative impact of their responses (114).

### 4.3 Treatment methods for psychological burden in cancer patients

Many cancer patients’ psychological problems are not timely discovered or intervened in. Jansen et al. pointed out that in the Netherlands, only 5%-9% of head and neck cancer patients used psychological care services at each time point from diagnosis to two years after treatment (115). Currently, there is no consensus on the management and treatment of psychological burden in cancer patients, but the mainstream methods can be roughly divided into hospital-level psychological therapy and social-level psychological care for cancer survivors. Systematic reviews indicate that the quality of clinical trials on the use of antidepressants for depression symptoms in cancer patients is poor, and there is no significant difference in efficacy between antidepressants and placebos in treating depression (116). Zetzl et al. introduced a yoga therapy program where breast cancer patients participated in an 8-week, 8-session yoga course. Compared to the control group, the yoga intervention group showed significantly reduced general fatigue (P = 0.033), physical fatigue (P = 0.048), and depression (P < 0.001), and significantly increased quality of life (P = 0.002) (117). Art therapy, which uses artistic expression (painting, music, etc.) as an intervention, has been reported to effectively reduce depression and anxiety symptoms in adult cancer patients, improving their quality of life (118). Kim et al.’s study found that breast cancer patients with mild to moderate depression who played a serious game (Hit the Cancer) showed reduced levels of depression compared to the control group, possibly because engaging in serious games that train attention promotes the normalization of functional connectivity in brain regions (119). Online cancer support groups (OCSGs), a product of the rapid development of computer networks and social media, connect cancer patients worldwide, providing information and emotional support to meet their psychological needs. Adikari et al. investigated the main times prostate cancer patients joined OCSGs and their changes in mental health. The results showed that patients joined the group at four phases: disease diagnosis, treatment, side effects, and recurrence. Except for patients with disease recurrence, negative emotional intensity significantly decreased in other group members (P < 0.005), with patients joining before treatment showing a more significant reduction in psychological burden (120). Additionally, interventions involving multidisciplinary teams, including oncologists, psychiatrists, nurses, and social workers, have been proven effective. The randomized controlled trial designed by Singer et al. showed that during hospitalization of cancer patients, interventions by multidisciplinary teams (including surgeons, nurses, psycho-oncologist, and social workers) reduced the frequency of financial problems (121). Dieperink et al.’s prospective clinical trial of prostate cancer patients divided them into a multidisciplinary intervention rehabilitation group and a control group, where the multidisciplinary group received two nursing consultation courses and guidance-oriented physical therapy in addition to routine care between 4 weeks and 6 months after radiotherapy. The study found that the intervention group showed higher fighting spirit than the control group six months after radiotherapy, and significantly lower cognitive avoidance three years later (122). Overall, in the future, clinicians should formulate treatment guidelines for the psychological burden of cancer patients based on larger-scale, more technologically advanced, and more rigorous clinical trials, to achieve multidisciplinary and multidimensional comprehensive management of the psychological burden in cancer patients.

## 5 Conclusion and outlook

Nowadays, psychological issues among cancer patients are becoming increasingly frequent. Cancer not only devastates the physical body but also shackles the mental world of patients. Fortunately, more and more healthcare professionals and social workers are starting to pay attention to the psychological burden of cancer patients. An increasing number of high-quality and rigorous clinical studies are being conducted to seek out the risk factors and effective intervention measures for the psychological burden of various types of cancer patients, aiming at early prediction and timely intervention of psychological issues. Compared to the general population, cancer patients have a significantly higher probability of experiencing psychological disorders such as depression, anxiety, demoralization syndrome, and adjustment disorders. In general, the psychological burden of cancer patients is related to multiple factors, including the type of cancer, cancer staging, the timing of cancer diagnosis, gender, age, and family income. However, there are currently many types of assessment scales for psychological issues, which are time-consuming to fill out. At the same time, there are various methods, including cognitive therapy, art therapy, and AI involvement, to intervene in the psychological burden of cancer patients. Still, the proportion of cancer patients with psychological issues who receive timely and effective psychological treatment is not as satisfactory as might be expected.

Therefore, in future research, considering the specificity of cancer types, researchers could leverage the advantages of machine learning to construct targeted predictive models for the psychological burden of cancer patients. Moreover, since patients often feel less ashamed discussing their conditions with machines compared to clinical physicians, this significant advantage can be utilized. Artificial intelligence can assess patients’ psychological health accurately and rapidly during their initial visits by evaluating objective parameters such as facial expressions, speech patterns, blood pressure, heart rate, and electroencephalograms (EEGs), thus minimizing the impact of subjective factors that may occur during self-assessment surveys and interviews. Apart from monitoring psychological issues in cancer patients, designing rigorous, larger sample size, and focused clinical trials on the prevention and intervention of psychological burden should also be implemented to achieve a comprehensive understanding and control of the psychological burden in cancer patients. With the cooperation of cancer patients, their families, oncology healthcare professionals, psychiatrists, and social workers, cancer patients will not only receive physical treatment but also more attention and assistance for their psychological burden.

## Author contributions

HW: Conceptualization, Methodology, Writing – original draft, Writing – review & editing. HL: Conceptualization, Data curation, Methodology, Writing – original draft, Writing – review & editing. BL: Conceptualization, Data curation, Investigation, Methodology, Supervision, Writing – original draft, Writing – review & editing.

## References

[1] Chhikara BS Parang K. Global Cancer Statistics 2022: the trends projection analysis. Chem Biol Lett. (2023) 10:451–1.

[2] Ju W Zheng R Zhang S Zeng H Sun K Wang S. Cancer statistics in Chinese older people, 2022: current burden, time trends, and comparisons with the US, Japan, and the Republic of Korea. Sci China Life Sci. (2023) 66:1079–91. doi: 10.1007/s11427-022-2218-x 36543994

[3] Siegel RL Miller KD Wagle NS Jemal A. Cancer statistics, 2023. CA Cancer J Clin. (2023) 73:17–48. doi: 10.3322/caac.21763 36633525

[4] Purushotham A Bains S Lewison G Szmukler G Sullivan R. Cancer and mental health—a clinical and research unmet need. Ann Oncol. (2013) 24:2274–8. doi: 10.1093/annonc/mdt214 23813928

[5] Lang-Rollin I Berberich G. Psycho-oncology. Dialogues Clin Neurosci. (2018) 20:13–22. doi: 10.31887/DCNS.2018.20.1/ilangrollin 29946207 PMC6016045

[6] Johannsen L Brandt M Frerichs W Inhestern L Bergelt C. The impact of cancer on the mental health of patients parenting minor children: a systematic review of quantitative evidence. Psychooncology. (2022) 31:869–78. doi: 10.1002/pon.5912 35218110

[7] Stark DPH House A. Anxiety in cancer patients. Br J Cancer. (2000) 83:1261–7. doi: 10.1054/bjoc.2000.1405 PMC2408796 11044347

[8] World Health Organization. The ICD-11 classification of mental and behavioral disorders: Clinical descriptions and diagnostic guidelines. Geneva: World Health Organization (2018).

[9] Bachem R Casey P. Adjustment disorder: a diagnosis whose time has come. J Affect Disord. (2018) 227:243–53. doi: 10.1016/j.jad.2017.10.034 29107817

[10] Harris BE Rice K Murray CV Thorsteinsson EB. Validation of the brief Adjustment Disorder New Modules with Australian oncology patients. BioPsychoSocial Med. (2023) 17:2. doi: 10.1186/s13030-022-00259-w PMC9875190 36698144

[11] Van Beek FE Wijnhoven LMA Custers JAE Holtmaat K De Rooij BH Horevoorts NJE. Adjustment disorder in cancer patients after treatment: prevalence and acceptance of psychological treatment. Supportive Care Cancer. (2021), 1–10. 10.1007/s00520-021-06530-0 PMC8486632 34599663

[12] Guze SB. Diagnostic and statistical manual of mental disorders, 4th ed. (DSM-IV). Am J Psychiatry. (1995) 152:8. doi: 10.1176/ajp.152.8.1228

[13] Kazlauskas E Patasius A Kvedaraite M Nomeikaite A Rudyte M Smailyte G. ICD-11 adjustment disorder following diagnostic procedures of prostate cancer: A 12-month follow-up study. J Psychosomatic Res. (2023) 168:111214. doi: 10.1016/j.jpsychores.2023.111214 36905705

[14] Dai D Coetzer H Zion SR Malecki MJ. Anxiety, depression, and stress reaction/adjustment disorders and their associations with healthcare resource utilization and costs among newly diagnosed patients with breast cancer. J Health Economics Outcomes Res. (2023) 10:68. doi: 10.36469/00001 PMC10062496 37008701

[15] Geue K Brähler E Faller H Härter M Schulz H Weis J. Prevalence of mental disorders and psychosocial distress in German adolescent and young adult cancer patients (AYA). Psycho-oncology. (2018) 27:1802–9. doi: 10.1002/pon.4730 29644783

[16] Naser AY Hameed AN Mustafa N Alwafi H Dahmash EZ Alyami HS. Depression and anxiety in patients with cancer: a cross-sectional study. Front Psychol. (2021) 12:1067. doi: 10.3389/fpsyg.2021.585534 PMC8081978 33935849

[17] Blázquez MH Cruzado JA. A longitudinal study on anxiety, depressive and adjustment disorder, suicide ideation and symptoms of emotional distress in patients with cancer undergoing radiotherapy. J psychosomatic Res. (2016) 87:14–21. doi: 10.1016/j.jpsychores.2016.05.010 27411747

[18] O’Donnell ML Agathos JA Metcalf O Gibson K Lau W. Adjustment disorder: Current developments and future directions. Int J Environ Res Public Health. (2019) 16:2537. doi: 10.3390/ijerph16142537 31315203 PMC6678970

[19] Soleimani MA Bahrami N Allen KA Alimoradi Z. Death anxiety in patients with cancer: A systematic review and meta-analysis. Eur J Oncol Nurs. (2020) 48:101803. doi: 10.1016/j.ejon.2020.101803 32836000

[20] Trill MD. Anxiety and sleep disorders in cancer patients. Eur J Cancer Suppl. (2013) 11:216–24. doi: 10.1016/j.ejcsup.2013.07.009 PMC4041166 26217130

[21] Wang YH Li JQ Shi JF Que JY Liu JJ Lappin JM. Depression and anxiety in relation to cancer incidence and mortality: a systematic review and meta-analysis of cohort studies. Mol Psychiatry. (2020) 25:1487–99. doi: 10.1038/s41380-019-0595-x 31745237

[22] Salvetti MDG MaChado CSP Donato SCT Silva AMD. Prevalence of symptoms and quality of life of cancer patients. Rev Bras Enfermagem. (2020) 73:e20180287. doi: 10.1590/0034-7167-2018-0287 32162649

[23] Götze H Friedrich M Taubenheim S Dietz A Lordick F Mehnert A. Depression and anxiety in long-term survivors 5 and 10 years after cancer diagnosis. Supportive Care Cancer. (2020) 28:211–20. 10.1007/s00520-019-04805-1 31001695

[24] Alagizy HA Soltan MR Soliman SS Hegazy NN Gohar SF. Anxiety, depression and perceived stress among breast cancer patients: single institute experience. Middle East Curr Psychiatry. (2020) 27:1–10. doi: 10.1186/s43045-020-00036-x

[25] Okati-Aliabad H Ansari-Moghadam A Mohammadi M Kargar S Shahraki-Sanavi F. The prevalence of anxiety and depression and its association with coping strategies, supportive care needs, and social support among women with breast cancer. Supportive Care Cancer. (2022) 30:703–10. doi: 10.1007/s00520-021-06477-2 34365523

[26] Breidenbach C Heidkamp P Hiltrop K Pfaff H Enders A Ernstmann N. Prevalence and determinants of anxiety and depression in long-term breast cancer survivors. BMC Psychiatry. (2022) 22:101. doi: 10.1186/s12888-022-03735-3 35139815 PMC8827186

[27] Yuan L Pan B Wang W Wang L Zhang X Gao Y. Prevalence and predictors of anxiety and depressive symptoms among patients diagnosed with oral cancer in China: a cross-sectional study. BMC Psychiatry. (2020) 20:1–15. doi: 10.1186/s12888-020-02796-6 32758185 PMC7405439

[28] Huang X Zhang TZ Li GH Liu L Xu GQ. Prevalence and correlation of anxiety and depression on the prognosis of postoperative non-small-cell lung cancer patients in North China. Medicine. (2020) 99. doi: 10.1097/MD.0000000000019087 PMC7440182 32176035

[29] Brunckhorst O Hashemi S Martin A George G Van Hemelrijck M Dasgupta P. Depression, anxiety, and suicidality in patients with prostate cancer: A systematic review and meta-analysis of observational studies. Prostate Cancer prostatic Dis. (2021) 24:281–9. doi: 10.1038/s41391-020-00286-0 32978524

[30] Clarke D Kissane D. Demoralization: its phenomenology and importance. Aust N Z J Psychiatry. (2002) 36:733–742. doi: 10.1046/j.1440-1614.2002.01086.x 12406115

[31] Tang L Li Z Pang Y. The differences and the relationship between demoralization and depression in Chinese cancer patients. Psycho-Oncology. (2020) 29:532–8. doi: 10.1002/pon.5296 31742784

[32] Lin CC Her YN. Demoralization in cancer survivors: an updated systematic review and meta-analysis for quantitative studies. Psychogeriatrics. (2024) 24:35–45. doi: 10.1111/psyg.13037 37877340

[33] Shao Q Li Y Lin L Boardman M Hamadi H Zhao M. Demoralization syndrome and its impact factors among cancer patients in China. J psychosocial Oncol. (2023), 1–16. 10.1080/07347332.2023.2249895 37609842

[34] Quintero Garzón L Koranyi S Engelmann D Philipp R Scheffold K Schulz‐Kindermann F. Perceived doctor-patient relationship and its association with demoralization in patients with advanced cancer. Psycho-oncology. (2018) 27:2587–93. 10.1002/pon.4823 29952046

[35] Bovero A Vitiello LP Botto R Gottardo F Cito A Geminiani GC. Demoralization in end-of-life cancer patients’ family caregivers: A cross-sectional study. Am J Hospice Palliative Medicine®. (2022) 39:332–9. doi: 10.1177/10499091211023482 34128389

[36] Momenimovahed Z Salehiniya H Hadavandsiri F Allahqoli L Günther V Alkatout I. Psychological distress among cancer patients during COVID-19 pandemic in the world: a systematic review. Front Psychol. (2021) 12:682154. doi: 10.3389/fpsyg.2021.682154 34650469 PMC8506116

[37] Wang Y Duan Z Ma Z Mao Y Li X Wilson A. Epidemiology of mental health problems among patients with cancer during COVID-19 pandemic. Trans Psychiatry. (2020) 10:263. doi: 10.1038/s41398-020-00950-y PMC7393344 32737292

[38] Bartmann C Fischer LM Hübner T Müller-Reiter M Wöckel A McNeill RV. The effects of the COVID-19 pandemic on psychological stress in breast cancer patients. BMC Cancer. (2021) 21:1–13. doi: 10.1186/s12885-021-09012-y 34972520 PMC8719114

[39] Romito F Dellino M Loseto G Opinto G Silvestris E Cormio C. Psychological distress in outpatients with lymphoma during the COVID-19 pandemic. Front Oncol. (2020) 10:1270. doi: 10.3389/fonc.2020.01270 32754447 PMC7365920

[40] Riedl D Schüßler G. Factors associated with and risk factors for depression in cancer patients–A systematic literature review. Trans Oncol. (2022) 16:101328. doi: 10.1016/j.tranon.2021.101328 PMC8741617 34990907

[41] Colombo N Lorusso D Scollo P. Impact of recurrence of ovarian cancer on quality of life and outlook for the future. Int J Gynecologic Cancer. (2017) 27. doi: 10.1097/IGC.0000000000001023 PMC5499966 28640766

[42] Anuk D Özkan M Kizir A Özkan S. The characteristics and risk factors for common psychiatric disorders in patients with cancer seeking help for mental health. BMC Psychiatry. (2019) 19:1–11. doi: 10.1186/s12888-019-2251-z 31481035 PMC6724340

[43] Rieke K Boilesen E Lydiatt W Schmid KK Houfek J Watanabe-Galloway S. Population-based retrospective study to investigate preexisting and new depression diagnosis among head and neck cancer patients. Cancer Epidemiol. (2016) 43:42–8. doi: 10.1016/j.canep.2016.06.008 27391545

[44] Manne SL Myers-Virtue S Kashy D Ozga M Kissane D Heckman C. Resilience, positive coping, and quality of life among women newly diagnosed with gynecological cancers. Cancer Nurs. (2015) 38:375–82. doi: 10.1097/NCC.0000000000000215 PMC4470889 25521911

[45] Hammermüller C Hinz A Dietz A Wichmann G Pirlich M Berger T. Depression, anxiety, fatigue, and quality of life in a large sample of patients suffering from head and neck cancer in comparison with the general population. BMC Cancer. (2021) 21:1–11. doi: 10.1186/s12885-020-07773-6 33482771 PMC7825198

[46] Wu HS Harden JK. Symptom burden and quality of life in survivorship: a review of the literature. Cancer Nurs. (2015) 38:E29–54. doi: 10.1097/NCC.0000000000000135 24831042

[47] Melissant HC Jansen F Eerenstein SE Cuijpers P Laan E Lissenberg-Witte BI. Body image distress in head and neck cancer patients: what are we looking at? Supportive Care Cancer. (2021) 29:2161–9. doi: 10.1007/s00520-020-05725-1 PMC7892513 32885315

[48] Lee JH Ba D Liu G Leslie D Zacharia BE Goyal N. Association of head and neck cancer with mental health disorders in a large insurance claims database. JAMA Otolaryngology–Head Neck Surg. (2019) 145:339–44. doi: 10.1001/jamaoto.2018.4512 PMC6481424 30816930

[49] Ravi P Karakiewicz PI Roghmann F Gandaglia G Choueiri TK Menon M. Mental health outcomes in elderly men with prostate cancer[C]//Urologic Oncology: Seminars and Original Investigations. Elsevier. (2014) 32:1333–40. doi: 10.1016/j.urolonc.2014.05.005 25153773

[50] Nead KT Sinha S Yang DD Nguyen PL. Association of androgen deprivation therapy and depression in the treatment of prostate cancer: a systematic review and meta-analysis[C]//Urologic Oncology: Seminars and Original Investigations. Elsevier. (2017) 35:664.e1–9. 10.1016/j.urolonc.2017.07.016 28803700

[51] Lima MP Longatto-Filho A Osório FL. Predictor variables and screening protocol for depressive and anxiety disorders in cancer outpatients. PloS One. (2016) 11:e0149421. doi: 10.1371/journal.pone.0149421 26954671 PMC4783052

[52] Oertelt-Prigione S de Rooij BH Mols F Oerlemans S Husson O Schoormans D. Sex-differences in symptoms and functioning in> 5000 cancer survivors: Results from the PROFILES registry. Eur J Cancer. (2021) 156:24–34. doi: 10.1016/j.ejca.2021.07.019 34411849

[53] Yilmaz M Dissiz G Usluoğlu AK Iriz S Demir F Alacacioglu A. Cancer-related stigma and depression in cancer patients in a middle-income country. Asia-Pacific J Oncol Nurs. (2020) 7:95–102. doi: 10.4103/apjon.apjon_45_19 PMC6927157 31879690

[54] Hong YR Yadav S Suk R Khanijahani A Erim D Turner K. Patient–provider discussion about emotional and social needs, mental health outcomes, and benefit finding among US Adults living with cancer. Cancer Med. (2021) 10:3622–34. doi: 10.1002/cam4.3918 PMC8178502 33960716

[55] Singer S Szalai C Briest S Brown A Dietz A Einenkel J. Co-morbid mental health conditions in cancer patients at working age–prevalence, risk profiles, and care uptake. Psycho-Oncology. (2013) 22:2291–7. doi: 10.1002/pon.3282 23494948

[56] Parpa E Tsilika E Gennimata V Mystakidou K. Elderly cancer patients’ psychopathology: a systematic review: aging and mental health. Arch gerontology geriatrics. (2015) 60:9–15. doi: 10.1016/j.archger.2014.09.008 25266607

[57] Eilami O Moslemirad M Naimi E Babuei A Rezaei K. The effect of religious psychotherapy emphasizing the importance of prayers on mental health and pain in cancer patients. J religion Health. (2019) 58:444–51. doi: 10.1007/s10943-018-0696-x 30225762

[58] Tsaras K Papathanasiou IV Mitsi D Veneti A Kelesi M Zyga S. Assessment of depression and anxiety in breast cancer patients: prevalence and associated factors. Asian Pacific J Cancer prevention: APJCP. (2018) 19:1661. 10.22034/APJCP.2018.19.6.1661 PMC6103579 29938451

[59] Ng GC Mohamed S Sulaiman AH Zainal NZ. Anxiety and depression in cancer patients: the association with religiosity and religious coping. J religion Health. (2017) 56:575–90. doi: 10.1007/s10943-016-0267-y 27287259

[60] Fradelos EC Latsou D Mitsi D Tsaras K Lekka D Lavdaniti M. Assessment of the relation between religiosity, mental health, and psychological resilience in breast cancer patients. Contemp Oncology/Współczesna Onkologia. (2018) 22:172–7. doi: 10.5114/wo.2018.78947 PMC6238091 30455589

[61] Bouras G Markar SR Burns EM Huddy JR Bottle A Athanasiou T. The psychological impact of symptoms related to esophagogastric cancer resection presenting in primary care: a national linked database study. Eur J Surg Oncol (EJSO). (2017) 43:454–60. doi: 10.1016/j.ejso.2016.10.010 27919514

[62] Saboonchi F Petersson LM Wennman-Larsen A Alexanderson K Brännström R Vaez M. Changes in caseness of anxiety and depression in breast cancer patients during the first year following surgery: patterns of transiency and severity of the distress response. Eur J Oncol Nurs. (2014) 18:598–604. doi: 10.1016/j.ejon.2014.06.007 24997517

[63] Lam WWT Soong I Yau TK Wong KY Tsang J Yeo W. The evolution of psychological distress trajectories in women diagnosed with advanced breast cancer: a longitudinal study. Psycho-Oncology. (2013) 22:2831–9. doi: 10.1002/pon.3361 24038545

[64] Liu Y Pettersson E Schandl A Markar S Johar A Lagergren P. Higher dispositional optimism predicts better health-related quality of life after esophageal cancer surgery: a nationwide population-based longitudinal study. Ann Surg Oncol. (2021) 28:7196–205. doi: 10.1245/s10434-021-10026-w PMC8521517 33876352

[65] Den Oudsten BL Van Heck GL van der Steeg AFW. Predictors of depressive symptoms 12 months after surgical treatment of early-stage breast cancer. Psycho-Oncology: J Psychological Soc Behav Dimensions Cancer. (2009) 18:1230–7. doi: 10.1002/pon.1518 19142843

[66] Usta YY. Importance of social support in cancer patients. Asian Pacific J Cancer Prev. (2012) 13:3569–72. doi: 10.7314/APJCP.2012.13.8.3569 23098436

[67] Song H Fang F Valdimarsdóttir U Lu D Andersson TM-L Hultman C. Waiting time for cancer treatment and mental health among patients with newly diagnosed esophageal or gastric cancer: a nationwide cohort study. BMC Cancer. (2017) 17:1–9. doi: 10.1186/s12885-016-3013-7 28049452 PMC5209901

[68] Lu W Pikhart H Peasey A Kubinova R Pitman A Bobak M. Risk of depressive symptoms before and after the first hospitalization for cancer: Evidence from a 16-year cohort study in the Czech Republic. J Affect Disord. (2020) 276:76–83. doi: 10.1016/j.jad.2020.06.070 32697719 PMC7456789

[69] Applebaum AJ Stein EM Lord-Bessen J Pessin H Rosenfeld B Breitbart W. Optimism, social support, and mental health outcomes in patients with advanced cancer. Psycho-oncology. (2014) 23:299–306. doi: 10.1002/pon.3418 24123339 PMC4001848

[70] Ladaninejad S Ilali E Mousavinasab N Taraghi Z. The relationship between depressive symptoms and demographic-medical characteristics among elder people with cancer. Asia-Pacific J Oncol Nurs. (2019) 6:424–30. doi: 10.4103/apjon.apjon_13_19 PMC6696808 31572764

[71] Hao A Huang J Xu X. Anxiety and depression in glioma patients: prevalence, risk factors, and their correlation with survival. Irish J Med Sci (1971-). (2021) 190:1155–64. doi: 10.1007/s11845-020-02374-5 33140294

[72] Zhou L Sun H. The longitudinal changes of anxiety and depression, their related risk factors and prognostic value in colorectal cancer survivors: a 36-month follow-up study. Clinics Res Hepatol Gastroenterol. (2021) 45:101511. doi: 10.1016/j.clinre.2020.07.016 33713979

[73] Alquraan L Alzoubi KH Rababa’h S Karasneh R Al-Azzam S Al-Azayzih A. Prevalence of depression and the quality-of-life of breast cancer patients in Jordan. J Multidiscip healthcare. (2020), 1455–62. doi: 10.2147/JMDH.S277243 PMC7650018 33177831

[74] Shi G Shi T Liu Y Cai Y. Relationships between dyadic coping, intimate relationship and post-traumatic growth in patients with breast cancer: A cross-sectional study. J Adv Nurs. (2021) 77:4733–42. doi: 10.1111/jan.14946 34227131

[75] Hann D Winter K Jacobsen P. Measurement of depressive symptoms in cancer patients: evaluation of the Center for Epidemiological Studies Depression Scale (CES-D). J psychosomatic Res. (1999) 46:437–43. doi: 10.1016/S0022-3999(99)00004-5 10404478

[76] Conerly RC Baker F Dye J Douglas CY Zabora J. Measuring depression in African American cancer survivors: The reliability and validity of the Center for Epidemiologic Study—Depression (CES-D) scale. J Health Psychol. (2002) 7:107–14. doi: 10.1177/1359105302007001658 22114231

[77] van Wilgen CP Dijkstra PU Stewart RE Ranchor AV Roodenburg JLN. Measuring somatic symptoms with the CES–D to assess depression in cancer patients after treatment: comparison among patients with oral/oropharyngeal, gynecological, colorectal, and breast cancer. Psychosomatics. (2006) 47:465–70. doi: 10.1176/appi.psy.47.6.465 17116946

[78] Lavdaniti M Govina OD Mylona E Prapa P-M Palitzika D Kosintzi A. CN64 Assessment of depression among lung cancer patients with type 2 diabetes using center for epidemiologic studies depression scale (CES-D). Ann Oncol. (2023) 34:S1244. doi: 10.1016/j.annonc.2023.09.1657

[79] Alzahrani AS Demiroz YY Alabdulwahab AS Alshareef RA Badri AS Alharbi BA. The diagnostic accuracy of the 9-item patient health questionnaire as a depression screening instrument in Arabic-speaking cancer patients. Neurology Psychiatry Brain Res. (2020) 37:110–5. doi: 10.1016/j.npbr.2020.07.003

[80] Degefa M Dubale B Bayouh F Ayele B Zewde Y. Validation of the PHQ-9 depression scale in Ethiopian cancer patients attending the oncology clinic at Tikur Anbessa specialized hospital. BMC Psychiatry. (2020) 20:1–7. doi: 10.1186/s12888-020-02850-3 32912183 PMC7488004

[81] Kroenke K Spitzer RL. The PHQ-9: a new depression diagnostic and severity measure. Psychiatr Ann. (2002) 32:509–15. doi: 10.3928/0048-5713-20020901-06

[82] Zigmond AS Snaith RP. The hospital anxiety and depression scale. Acta psychiatrica scandinavica. (1983) 67:361–70. doi: 10.1111/j.1600-0447.1983.tb09716.x 6880820

[83] Wondie Y Mehnert A Hinz A. The hospital anxiety and depression scale (HADS) applied to Ethiopian cancer patients. PloS One. (2020) 15:e0243357. doi: 10.1371/journal.pone.0243357 33270779 PMC7714130

[84] Skarstein J Aass N Fosså SD Skovlund E Dahl AA. Anxiety and depression in cancer patients: relation between the Hospital Anxiety and Depression Scale and the European Organization for Research and Treatment of Cancer Core Quality of Life Questionnaire. J psychosomatic Res. (2000) 49:27–34. doi: 10.1016/S0022-3999(00)00080-5 11053601

[85] Richter P Werner J Heerlein A Kraus A Sauer H. On the validity of the Beck Depression Inventory: A review. Psychopathology. (1998) 31:160–8. doi: 10.1159/000066239 9636945

[86] Almeida S Camacho M Barahona-Corrêa JB Oliveira J Lemos R da Silva DR. Criterion and construct validity of the Beck Depression Inventory (BDI-II) to measure depression in patients with cancer: The contribution of somatic items. Int J Clin Health Psychol. (2023) 23:100350. doi: 10.1016/j.ijchp.2022.100350 36467263 PMC9709241

[87] Bener A Alsulaiman R Doodson LG El Ayoubi HR. Comparison of Reliability and Validity of the Breast Cancer depression anxiety stress scales (DASS-21) with the Beck Depression Inventory-(BDI-II) and Hospital Anxiety and Depression Scale (HADS). Int J Behav Res Psychol. (2016) 4:197–203.

[88] Fox RS Lillis TA Gerhart J Hoerger M Duberstein P. Multiple group confirmatory factor analysis of the DASS-21 depression and anxiety scales: how do they perform in a cancer sample? psychol Rep. (2018) 121:548–65. doi: 10.1177/0033294117727747 PMC6053047 28836917

[89] Harms J Kunzmann B Bredereke J Harms L Jungbluth T Zimmermann T. Anxiety in patients with gastrointestinal cancer undergoing primary surgery. J Cancer Res Clin Oncol. (2023), 1–10. doi: 10.1007/s00432-023-04759-2 PMC10374702 37060473

[90] Vaioulis A Bonotis K Perivoliotis K Kiouvrekis Y Gravas S Tzortzis V. Quality of life and anxiety in patients with first diagnosed non-muscle invasive bladder cancer who receive adjuvant bladder therapy. Bladder Cancer. (2021) 7:297–306. doi: 10.3233/BLC-201524 PMC11181705 38993613

[91] Grilo AM Gomes AI Monsanto F Albino D Augusto C Pragana C. First day of radiotherapy for women with breast cancer: predictors of anxiety. Supportive Care Cancer. (2020) 28:1241–8. doi: 10.1007/s00520-019-04902-1 31227988

[92] Koranyi S Hinz A Hufeld JM Hartung TJ Garzón LQ Fendel U. Psychometric evaluation of the German version of the demoralization scale-II and the association between demoralization, sociodemographic, disease-and treatment-related factors in patients with cancer. Front Psychol. (2021) 12:789793. doi: 10.3389/fpsyg.2021.789793 34899543 PMC8652041

[93] Belar A Arantzamendi M Rodríguez-Núñez A Santesteban Y Martinez M López-Saca M. Multicenter study of the psychometric properties of the new Demoralization Scale (DS-II) in Spanish-speaking advanced cancer patients. J Pain Symptom Manage. (2019) 57:627–34. doi: 10.1016/j.jpainsymman.2018.11.016 30472315

[94] Wu W-J Quan M-M Gao L Li Q Yan C-X Zhang Q. Demoralization and depression in Chinese cancer patients. Supportive Care Cancer. (2021) 29:6211–6. doi: 10.1007/s00520-021-06195-9 33834301

[95] Chrobak AA Przydacz M Chłosta M Machalska K Turek A Popiół M. Bipolar spectrum in prostate cancer patients and its role in stress related symptoms. Psycho-Oncology. (2023) 32:438–45. doi: 10.1002/pon.6096 36631917

[96] Hahn EE Hays RD Kahn KL Litwin MS Ganz PA. Post-traumatic stress symptoms in cancer survivors: relationship to the impact of cancer scale and other associated risk factors. Psycho-Oncology. (2015) 24:643–52. doi: 10.1002/pon.3623 PMC4400255 25059888

[97] Caviness-Ashe N Zimmerman S Chappel-Aiken L Onsomu EO Bryant AL Smith SK. Exploring the relationship between social support and mental health status among lymphoma survivors: Does patient-centered communication really matter? A brief report. J Psychosocial Oncol. (2023) 41:235–41. doi: 10.1080/07347332.2022.2072792 PMC9971635 36815246

[98] Banienė I Žemaitienė N. Post-traumatic stress symptoms among Lithuanian parents raising children with cancer. Children. (2020) 7:116. doi: 10.3390/children7090116 32878191 PMC7552768

[99] Creamer M Bell R Failla S. Psychometric properties of the impact of event scale - revised. Behav Res Ther. (2003) 41:1489–96. doi: 10.1016/j.brat.2003.07.010 14705607

[100] Beeber LS Shea J McCorkle R. The Center for Epidemiology Studies Depression Scale as a measure of depressive symptoms in newly diagnosed patients. J psychosocial Oncol. (1998) 16:1–20. doi: 10.1300/J077V16N01_01

[101] Bergua V Meillon C Potvin O Ritchie K Tzourio C Bouisson J. Short STAI-Y anxiety scales: validation and normative data for elderly subjects. Aging Ment Health. (2016) 20:987–95. doi: 10.1080/13607863.2015.1051511 26055726

[102] Wu Y Levis B Sun Y He C Krishnan A Neupane D. Accuracy of the Hospital Anxiety and Depression Scale Depression subscale (HADS-D) to screen for major depression: systematic review and individual participant data meta-analysis. bmj. (2021) 373. doi: 10.1136/bmj.n972 PMC8107836 33972268

[103] Annunziata MA Muzzatti B Bidoli E Flaiban C Bomben F Piccinin M. Hospital Anxiety and Depression Scale (HADS) accuracy in cancer patients. Supportive Care Cancer. (2020) 28:3921–6. doi: 10.1007/s00520-019-05244-8 31858249

[104] Hartung TJ Friedrich M Johansen C Wittchen H-U Faller H Koch U. The Hospital Anxiety and Depression Scale (HADS) and the 9-item Patient Health Questionnaire (PHQ-9) as screening instruments for depression in patients with cancer. Cancer. (2017) 123:4236–43. doi: 10.1002/cncr.30846 28654189

[105] Newport DJ Nemeroff CB. Assessment and treatment of depression in the cancer patient. J psychosomatic Res. (1998) 45:215–37. doi: 10.1016/S0022-3999(98)00011-7 9776368

[106] Niedzwiedz CL Knifton L Robb KA Katikireddi SV Smith DJ. Depression and anxiety among people living with and beyond cancer: a growing clinical and research priority. BMC Cancer. (2019) 19:1–8. doi: 10.1186/s12885-019-6181-4 31604468 PMC6788022

[107] Kodipalli A Devi S. Analysis of fuzzy based intelligent health care application system for the diagnosis of mental health in women with ovarian cancer using computational models. Intelligent Decision Technol. (2023) 17:31–42. doi: 10.3233/IDT-228006

[108] Smrke U Mlakar I Lin S Musil B Plohl N. Language, speech, and facial expression features for artificial intelligence–based detection of cancer survivors’ depression: scoping meta-review. JMIR Ment Health. (2021) 8:e30439. doi: 10.2196/30439 34874883 PMC8691410

[109] Chen L. Facial expression recognition with machine learning and assessment of distress in patients with cancer. Number 1/January 2021. (2021) 48:81–93. doi: 10.1188/21.ONF.81-93 33337433

[110] Zhang A Kamat A Acquati C Aratow M Kim JS DuVall AS. Evaluating the feasibility and acceptability of an artificial-intelligence-enabled and speech-based distress screening mobile app for adolescents and young adults diagnosed with cancer: A study protocol. Cancers. (2022) 14:914. doi: 10.3390/cancers14040914 35205663 PMC8870320

[111] Li F Sun H Biswal BB Sweeney JA Gong Q. Artificial intelligence applications in psychoradiology. Psychoradiology. (2021) 1:94–107. doi: 10.1093/psyrad/kkab009 37881257 PMC10594695

[112] Liu G-D Li Y-C Zhang W Zhang L. A brief review of artificial intelligence applications and algorithms for psychiatric disorders. Engineering. (2020) 6:462–7. doi: 10.1016/j.eng.2019.06.008

[113] Zhang W Zou Y Zhao F Yang Y Mao N Li Y. Brain network alterations in rectal cancer survivors with depression tendency: evaluation with multimodal magnetic resonance imaging. Front Neurol. (2022) 13:791298. doi: 10.3389/fneur.2022.791298 35847225 PMC9277124

[114] Lucas GM Gratch J King A Morency L-P. It’s only a computer: Virtual humans increase willingness to disclose. Comput Hum Behav. (2014) 37:94–100. doi: 10.1016/j.chb.2014.04.043

[115] Jansen F Lissenberg-Witte BI Hardillo JA Takes RP de Bree R Lamers F. Mental healthcare utilization among head and neck cancer patients: A longitudinal cohort study. Psycho-Oncology. (2024) 33:e6251. doi: 10.1002/pon.6251 37955598

[116] Ostuzzi G Matcham F Dauchy S Barbui C Hotopf M. Antidepressants for the treatment of depression in people with cancer. Cochrane Database Systematic Rev. (2018) 4). doi: 10.1002/14651858.CD011006.pub3 PMC6494588 29683474

[117] Zetzl T Renner A Pittig A Jentschke E Roch C van Oorschot B. Yoga effectively reduces fatigue and symptoms of depression in patients with different types of cancer. Supportive Care Cancer. (2021) 29:2973–82. doi: 10.1007/s00520-020-05794-2 PMC8062403 33026490

[118] Bosman JT Bood ZM Scherer-Rath M Dörr H Christophe N Sprangers MAG. The effects of art therapy on anxiety, depression, and quality of life in adults with cancer: a systematic literature review. Supportive Care Cancer. (2021) 29:2289–98. doi: 10.1007/s00520-020-05869-0 PMC7981299 33188476

[119] Kim SM Kim H-J Hwang HC Hong JS Bae S Min KJ. The effects of a serious game on depressive symptoms and anxiety in breast cancer patients with depression: a pilot study using functional magnetic resonance imaging. Games Health J. (2018) 7:409–17. doi: 10.1089/g4h.2017.0183 30383458

[120] Adikari A de Silva D Ranasinghe WKB Bandaragoda T Alahakoon O Persad R. Can online support groups address psychological morbidity of cancer patients? An artificial intelligence based investigation of prostate cancer trajectories. PloS One. (2020) 15:e0229361. doi: 10.1371/journal.pone.0229361 32130256 PMC7055800

[121] Singer S Roick J Meixensberger J Schiefke F Briest S Dietz A. The effects of multi-disciplinary psycho-social care on socio-economic problems in cancer patients: a cluster-randomized trial. Supportive Care Cancer. (2018) 26:1851–9. doi: 10.1007/s00520-017-4024-x 29270828

[122] Dieperink KB Johansen C Hansen S Wagner L Andersen KK Minet LR. Male coping through a long-term cancer trajectory. Secondary outcomes from a RTC examining the effect of a multidisciplinary rehabilitation program (RePCa) among radiated men with prostate cancer. Acta Oncol. (2017) 56:254–61. doi: 10.1080/0284186X.2016.1267395 28093012

